# Editorial: Computational Approaches in Drug Discovery and Precision Medicine

**DOI:** 10.3389/fchem.2020.639449

**Published:** 2021-02-12

**Authors:** Zunnan Huang, Xiao Jun Yao, Ruo-Xu Gu

**Affiliations:** ^1^ Key Laboratory of Big Data Mining and Precision Drug Design of Guangdong Medical University, Guangdong Medical University, Dongguan, China; ^2^ Dr. Neher's Biophysics Laboratory for Innovative Drug Discovery, Macau University of Science and Technology, Macau, China; ^3^ Department of Theoretical and Computational Biophysics, Max-Planck Institute for Biophysical Chemistry, Göttingen, Germany

**Keywords:** drug discovery, precision medicine, molecular modeling, data mining, artificial intelligence

During the past decades, computational approaches have been highly involved in all stages of drug discovery (Terstappen and Reggiani, 2001; Duarte et al., 2019) and precision medicine (Barbolosi et al., 2016; Delavan et al., 2018), from screening of leading compounds to preclinical trials. These methods, including both structure-based molecular modeling techniques (Kalyaanamoorthy and Chen, 2011) and artificial intelligence (Fleming, 2018; Williams et al., 2018; Chan et al., 2019), accelerate the discovery of drug candidates, guide the repurposing of existing drugs, improve our understanding of biomolecular nanomachines, and reduce the use of experimental animals. To present state-of-the-art computational studies in this field, we launched a research topic in *Frontiers in Chemistry* entitled “*Computational Approaches in Drug Discovery and Precision Medicine*.” This research topic included nine articles, including two reviews and seven original research articles, which covered theoretical predictions of ligand–protein interactions, drug resistance mechanism, and drug selectivity mechanism in protein–ligand binding, and the prediction of preclinical properties of ligands as well.

Two manuscripts reported case study of virtual screening of drug candidates. Han et al.constructed natural product database by analyzing the ingredients of traditional Chinese medicine prescriptions for treating prostate cancer. Molecular docking and wet experiments were then performed to screen possible ligands targeting androgen receptor, a protein involved in the pathogenesis of prostate cancer. Halim et al. docked active components of frankincense, macrocyclic diterpenoid derivatives, and boswellic acids to multiple proteins which are known drug targets of psoriasis in order to screen possible candidates for treating psoriasis. In addition, the review article by Zhao et al. summarized recent structure-based and ligand-based virtual screening of analgesics targeting opioid receptors, the computational guided studies of subtype selectivity of *σ* and *κ* opioid receptors, as well as the activation mechanism.

Molecular modeling has become an important complimentary to experiments in the study of ligand–protein interaction mechanisms. In this regard, Yang et al. investigated subtype selectivity mechanism of pyrabactin, an abscisic acid (ABA)-mimicking ligand, for the ABA receptors, using sequence and structural comparison and free energy calculations. Zhang et al. explored how the mutations of *Mycobacterium tuberculosis* RNA polymerase develop drug resistance to rifampicin.

During the past years, artificial intelligence has been increasingly involved in drug development. In this research topic, two manuscripts employed the application of machine learning to predict the ligand–protein interactions. Zhang et al. proposed a method to represent the SMILES strings of ligands and protein sequences to lower-dimensional vectors, which revealed the hidden biophysical and biochemical patterns of ligands and proteins. Their methods are able to predict drug–target interactions in an efficient and reliable way, and will be helpful for repurposing of drugs. Li et al. developed a deep learning–based technique to predict the association between drugs and diseases, using the information of molecular structures and disease symptoms.

One reason for the failure of drug development is undesired pharmacokinetics and toxicity of the ligands. *In silico* evaluation of the ADMET properties of preclinical drugs is able to reduce the cost of drug development significantly. Wu et al. systematically reviewed the current methods, databases, and software developed for the *in vitro* prediction of ADMET properties. They also described related application of these methods, discussed the challenges and limitations of this field, and proposed suggestions.

Molecular dynamics simulation has been an effective tool to study the mechanism of the biological function of proteins. Bai et al. investigate the conformational transition of glucagon receptor (GCGR) in the apo, glucagon bound, and antagonist bound forms by conventional and accelerated molecular dynamics simulations. Their work provided an example of how different ligands modulate the conformational transition of a protein between different states, and proved that accelerated molecular dynamics simulation is a reliable way to explore the conformational space of proteins.

To summarize, this research topic provides the readers an overview of how computational methods accelerate drug development and increase our understanding of ligand–protein interactions. We sincerely hope the articles in this research topic can draw people’s attention and promote the researches in the field of computational studies in drug discovery and precision medicine.
